# Proximal Roux-en-y Gastrojejunal Anastomosis with Pyloric Ring Resection Improves Gastric Emptying After Pancreaticoduodenectomy

**DOI:** 10.1007/s11605-016-3091-5

**Published:** 2016-02-05

**Authors:** Omar Barakat, Martha N. Cagigas, Shima Bozorgui, Claire F. Ozaki, R. Patrick Wood

**Affiliations:** Department of Hepatobiliary and Pancreatic Surgery, CHI St. Luke’s Health–Baylor St. Luke’s Medical Center, 6624 Fannin, Suite 2180, Houston, TX 77030 USA

**Keywords:** Delayed gastric emptying, Whipple, Motilin, Pancreatic cancer

## Abstract

**Background:**

Delayed gastric emptying (DGE) is a common complication of pancreaticoduodenectomy. We determined the efficiency of a new reconstruction technique, designed to preserve motilin-secreting cells and maximize the utility of their receptors, in reducing the incidence of DGE after pancreaticoduodenectomy.

**Methods:**

From April 2005 to September 2014, 217 consecutive patients underwent pancreaticoduodenectomy at our institution. Nine patients who underwent total pancreatectomy were excluded. We compared outcomes between patients who underwent pancreaticoduodenectomy with resection of the pyloric ring followed by proximal Roux-en-y gastrojejunal anastomosis (group I, *n* = 90) and patients who underwent standard pancreaticoduodenectomy with the orthotopic reconstruction technique (group II, *n* = 118).

**Results:**

Overall and clinically relevant rates of DGE were significantly lower in group I than in group II (10 and 2.2 % vs. 57 and 24 %, respectively; *p* < 0.05). Length of hospital stay as a result of DGE was shorter in group I than in group II. In univariate analysis, older age, comorbidities, ASA grade 4, operative time, preoperative diabetes, standard reconstruction technique, and postoperative complications were significant risk factors for DGE. In multivariate analysis, older age, standard technique, and postoperative complications were independent risk factors for DGE.

**Conclusion:**

Our new reconstruction technique reduces the occurrence of DGE after pancreaticoduodenectomy.

## Introduction

Delayed gastric emptying (DGE), a common complication of pancreaticoduodenectomy (PD), has an incidence ranging from 3 to 61 %, depending on the reconstruction technique and definition used.^1^–^19^ Although DGE is non-fatal and self-limiting, it contributes significantly to increased patient discomfort, hospital length of stay, and medical costs. The pathogenesis of DGE after PD is multifactorial. Although perioperative risk factors and postoperative intra-abdominal complications contribute to the overall incidence of DGE, the disruption of neuro-humoral pathways remains the common denominator in all patients after PD that contributes to the occurrence of primary DGE.

Gastric motility is a coordinated process of myoelectrical activities involving the gastric reservoir, antral pump, pylorus, and duodenum. These activities are controlled by both entero-gastric neural reflexes and hormones released from the intestines that reach the stomach via the systemic circulation. Gastric emptying occurs during two distinct periods: the postprandial motility period that starts after each meal and the subsequent interdigestive period, during which the gastrointestinal tract produces rhythmically recurring cycles of activity that involve four phases. Phase III, known as the migrating motor complex (MMC), consists of forceful peristaltic waves that originate simultaneously in the stomach and the duodenum and propagate along the entire length of the small intestine. The main function of the MMC is to empty the stomach and small intestine from chyme residues, mucous, and bacteria.^20^–^22^ The recurrence of phase III at the end of the postprandial period is facilitated by the release of motilin hormone secreted from endocrine cells in the gastric antrum and duodenum.^23^ Motilin then reaches the stomach and small intestine via the systemic circulation, acting on receptors found at high concentrations on the smooth muscle and nerve endings in the gastric antrum, duodenum, and proximal jejunum.^9^,^23^,^24^ As the MMC activity reaches the small intestine, the velocity of propagation of the peristaltic waves declines from the proximal jejunum to the distal small intestine, which is most likely related to a decreased number of motilin receptors along the gastrointestinal tract.^20^

Standard PD entails the removal of the gastric antrum, head of the pancreas, duodenum, common bile duct, and the proximal 5 to 10 cm of jejunum. The magnitude of the resection and reconstruction disrupts the aforementioned neuro-humoral pathways that coordinate the myoelectrical activities responsible for gastric emptying.^25^–^27^ Impaired postprandial motor activity after PD has been explained by a significant reduction in levels of circulating gastrin and other gastrointestinal hormones normally secreted by the stomach and the duodenum.^28^ Furthermore, resection of the gastric antrum and duodenum has been shown to reduce the cyclic increase in plasma levels of motilin, thus impairing phase III activity in the efferent loop of the gastrojejunal anastomosis (GJA).^29^ However, effective stimulation of gastric emptying after PD can be achieved by a motilin receptor agonist such as erythromycin, which has been shown to enhance phase III gastric contraction via motilin receptors.^19^,^30^

Different techniques for GJA have been proposed to improve gastric emptying after PD.^9^,^10^,^12^,^31^–^34^ Pylorus-preserving PD (PpPD) was developed and widely used to improve patients’ nutrition and to reduce the complications related to standard PD.^35^–^37^ However, abnormalities of postprandial and MMC activities involving the afferent and efferent limb of the GJA, together with low amplitude and frequency, have been documented in up 70 % of patients after either standard PD or PpPD.^29^,^38^ Currently, no conspicuous technique has been described that can explicitly eliminate the incidence of primary DGE. The majority of techniques are used to construct the GJA on the first loop of the jejunum 25 to 30 cm distal to the pancreatojejunal (PJ) and hepatojejunal (HJ) anastomosis. Because the concentration of motilin receptors and the velocity of propagation of MMC activity decline from the proximal to the distal small intestine, we hypothesized that the effective stimulation of gastric emptying after PD may be achieved by preserving the gastric antrum and utilizing the proximal end of the first jejunal loop for reconstruction with the gastric antrum, followed by distal Roux-en-y PJ and HJ anastomosis.

In this prospective cohort pilot study, we examined whether the preservation of the gastric antrum with proximal Roux-en-y GJA reduces the incidence of DGE when compared to standard PD using the orthotopic reconstruction technique.

## Materials and Methods

### Patients and Study Design

Between April 2005 and September 2014, 217 consecutive patients underwent elective PD for benign and malignant periampullary diseases at our institution. Nine of the 217 patients who underwent total pancreatectomy were excluded from the study. Of the 208 patients who were included in the study, 90 were prospectively studied patients who underwent PD with the new technique from June 2011 to September 2014 (group I); the other 118 patients were historical patients who underwent standard PD with orthotopic reconstruction from April 2005 to June 2011 (group II). Data analysis was performed according to a protocol approved by the CHI St. Luke’s–Baylor St. Luke’s Medical Center Institutional Review Board. Results were compared between group I and group II.

Preoperative data included age, sex, American Society of Anesthesiologists (ASA) grade, preoperative serum albumin and total bilirubin levels, and the presence of preoperative biliary drainage, diabetes, and comorbidity (Table 1). Perioperative data included operative time, estimated blood loss, perioperative blood transfusion, type of PD, concomitant procedures including portal vein resection, and presence of biliary infection (Table 1). Postoperative outcomes included overall surgery-related complications other than DGE, serum glucose level, pathologic diagnosis, hospital length of stay, 30-day readmission rate, 90-day mortality rate (Table 2), and the overall incidence of DGE (Table 3).

**Table 1:** Preoperative and intraoperative variables in group I and group II

Variables	Group I (*n* = 90)	Group II (*n* = 118)	*p* value
Preoperative variables			
Age	62 (30–85)	62 (24–84)	0.62
Sex			0.77
Male	42 (46.7 %)	58 (49.2 %)	
Female	48 (53.3 %)	60 (51.0 %)	
ASA grade			0.36
2	26 (28.9 %)	35 (29.7 %)	
3	58 (64.4 %)	67 (56.8 %)	
4	6 (6.7 %)	16 (13.6 %)	
ERCP/PTC	66 (73.3 %)	76 (64.4 %)	0.27
Diabetes	33 (36.7 %)	33 (28.0 %)	0.045*
Other comorbidity	30 (33.3 %)	44 (37.3 %)	0.25
Serum albumin (g/dl)	4.1 (1.6–4.8)	4.2 (2.1–5.3)	0.59
Total bilirubin (mg/dl)	0.6 (0.2–29.7)	0.8 (0.2–18.3)	0.45
Intraoperative variables			
Operative time (min)	347 (197–477)	391 (227–885)	<0.001*
Estimated blood loss (ml)	181 (25–1900)	300 (50–3200)	0.53
Blood transfusion	14 (15.6 %)	29 (24.6 %)	0.41
Type of PD			0.15
Standard	90 (100 %)	116 (98.3 %)	
Pylorus preserving	0	2 (1.7 %)	
Concomitant procedure	10 (11.1 %)	10 (8.5 %)	0.56
Biliary infection	66 (73.3 %)	76 (64.4 %)	0.27

Data are expressed as the median (range) or as the number (percent)

*ASA* American Society of Anesthesiologists, *ERCP/PTC* endoscopic retrograde cholangiopancreatography/percutaneous transhepatic cholangiography, *PD* pancreaticoduodenectomy

*p < 0.05 was considered statistically significant

**Table 2:** Postoperative outcomes in group I and group II

Variable	Group 1 (*n* = 90)	Group II (*n* = 118)	*p* value
Serum glucose (mg/dl)	133 (87–244)	152 (98–445)	<0.001*
Complications other than DGE and POPF	17 (18.9 %)	57 (48.3 %)	0.03*
POPF			0.78
Overall	6 (6.7 %)	11 (9.3 %)	
Clinically relevant (Grades B and C)	3 (3.3 %)	5 (4.2 %)	
Underlying disease			0.57
Malignant	65 (72.2 %)	84 (71.2 %)	
Benign	25 (27.8 %)	34 (28.8 %)	
Length of hospital stay	6 days	11 days	<0.001*
Overall 30-day readmission	17/88 (19.3 %)	32/114 (28.1 %)	0.94
90-day mortality	2 (2.2 %)	4 (3.4 %)	0.82

Data are expressed as the median (range) or as the number (percent)

*DGE* delayed gastric emptying, *POPF* postoperative pancreatic fistula

*p < 0.05 was considered statistically significant

**Table 3:** Overall complications classified by severity

Grade	Overall (*n*)	Group I (*n*)	Group II (*n*)
I	5	2	3
II	46	15	31
IIIa	35	5	30
IIIb	1	0	1
IVa	4	1	3
IVb	0	0	0
Total	91	23	68*

*p = 0.03 for group I vs. group II

Postoperative complications were graded by severity according to the classification system adopted for pancreatic surgery, which relies on the type of treatment used for each complication.^39^ Grade I complications included any deviation from the normal postoperative course not requiring pharmacologic treatment or intervention. Grade II complications required pharmacologic treatment such as blood transfusion or total parenteral nutrition. Grade III complications required interventional treatment without general anesthesia (IIIa) or with general anesthesia (IIIb). Grade IV complications were life-threatening complications that required ICU management. Postoperative pancreatic fistula (POPF) was classified as grade A, B, or C according to the International Study Group on Pancreatic Fistula (ISGPF).^40^ Grade A was defined as the leakage of fluid with high amylase content that had no clinical impact and that resolved spontaneously with conservative management. Clinically relevant POPF (grades B and C) was defined as the leakage of fluid in association with fever (38 °C), leukocytosis, and the need for therapeutic intervention.

The primary study end point was the rate and grade of DGE. The secondary end points were factors contributing to DGE, hospital length of stay, and readmission rate related to DGE.

### Surgical Technique

**Fig. 1:**
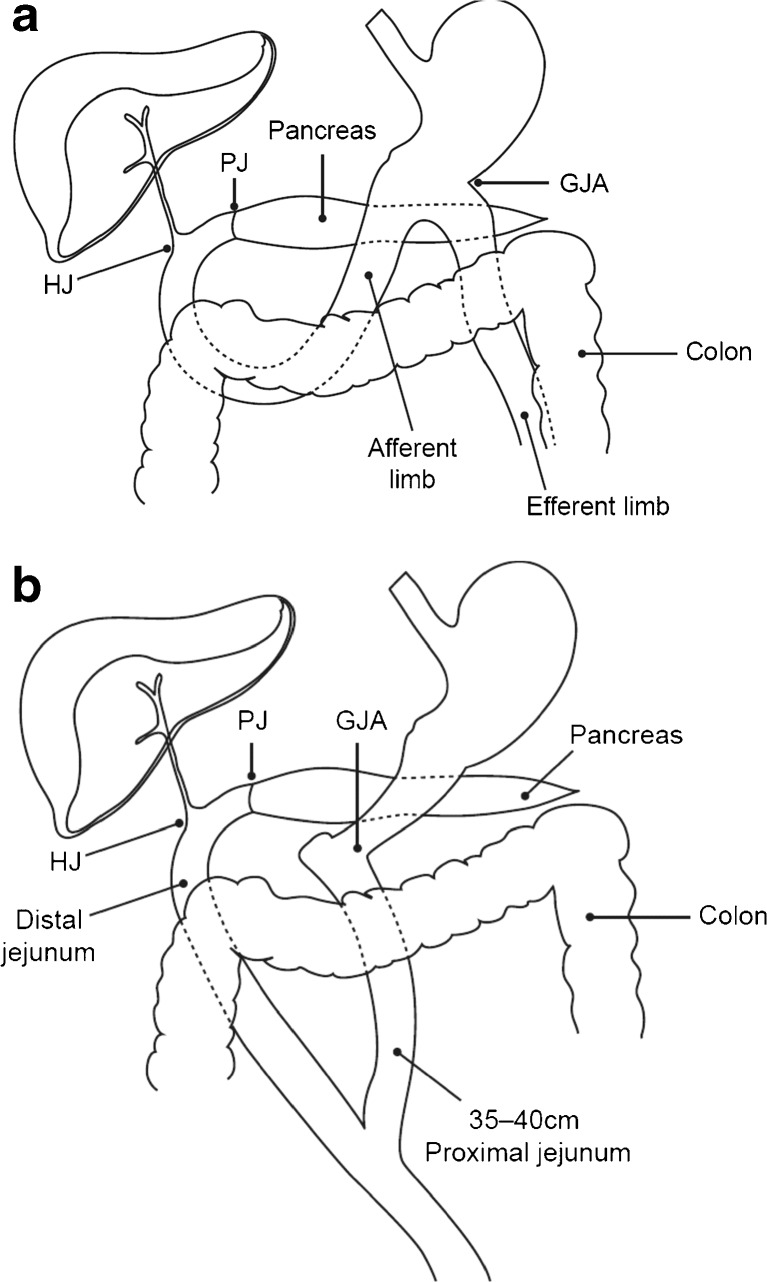
Schematic illustrations showing (**a**) standard pancreaticoduodenectomy (PD) with orthotopic reconstruction (group II patients) and (**b**) PD with the new reconstruction technique (group I patients), which utilizes the proximal 35 to 40 cm of the jejunum for the gastrojejunal anastomosis (GJA) and the distal loop for the pancreatojejunal (PJ) and hepatojejunal (HJ) anastomosis. Both anastomoses are placed in a retrocolic position

In all group II patients, standard PD with antrectomy was the procedure of choice. The extent of lymph node dissection was performed according to the underlying disease. In patients with a benign or low-grade malignancy, such as chronic pancreatitis or intraductal papillary mucinous neoplasm, we performed D1 lymph node dissection (i.e., dissection of peripancreatic head lymph nodes). In patients with malignant disease, we performed D2 lymph node dissection (i.e., dissection of lymph nodes along the hepatoduodenal ligament, common hepatic artery, portal caval, and peripyloric region). Dissection of the lymph nodes along the superior mesenteric artery was preserved for tumor involving the uncinate process. After completion of the resection, reconstruction was performed by using the orthotopic approach shown in Fig. 1a, in which the proximal jejunal loop was rotated behind the root of the mesentery and placed within the duodenal bed. After completion of the pancreatojejunal anastomosis (PJA) and hepatojejunal anastomosis (HJA), a hand-sewn, isoperistaltic GJA was performed 25 to 30 cm distal to the HJA in two layers by using 3–0 polydioxanone (PDS) sutures and 4–0 silk sutures (Fig. 1a).

In group I patients who underwent PD with the new technique, the distal antrum was divided about 1 to 2 cm proximal to the pylorus ring, preserving more than 95 % of the stomach. The proximal jejunum was divided approximately 2 to 4 cm distal to the duodeno-jejunal junction. After completion of the resection, the proximal end of the first loop of jejunum was then brought through the transverse mesocolon, and the GJA was performed in two layers by using 3–0 PDS sutures and 4–0 silk sutures in an end-to-side fashion. The jejunum was then divided 35 to 40 cm distal to the GJA, and the distal limb was brought separately through the transverse mesocolon to be placed in the duodenal bed for reconstruction of the PJA and HJA (Fig. 1b). The two loops of jejunum were then anastomosed caudal to the transverse mesocolon by using a gastrointestinal stapling device.

In both groups, the PJA and HJA were completed in a similar fashion, as described previously.^41^ Briefly, an end-to-side, duct-to-mucosa, two-layer PJA was performed by using interrupted, non-absorbable prolene sutures. Depending on the size of the pancreatic duct, four to six 6–0 prolene sutures were placed within the pancreatic duct before placement of the posterior outer-layer sutures. The posterior outer-layer anastomosis was performed by using a horizontal mattress technique between the whole thickness of the pancreatic remnant and the seromuscular layer of the jejunum with 4–0 or 5–0 prolene sutures. Upon completion of the posterior outer layer, the duct-to-mucosa anastomosis was then completed. In patients with pancreatic duct diameter ≤3 mm, the anastomosis was performed over a 6-cm 5-F internal plastic stent (Kendall Argyle feeding tube, Tyco Healthcare, Mansfield, MA). The anterior outer layer of the anastomosis was then completed between the pancreas and the seromuscular layer of the jejunum by using 5–0 prolene sutures. After completion of the PJA, an end-to-side HJA was performed by using interrupted 5–0 PDS without a stent, approximately 5 to 10 cm distal to the PJA. A feeding jejunostomy tube was not placed. In all cases, two Jackson-Pratt silicon drains were introduced through separate stab incisions and were placed anterior and posterior to the PJA.

All procedures were performed by one senior hepatobiliary and pancreatic surgeon (OB) and assisted by two other senior surgeons (CFO, RPW).

### Perioperative Management

Following the procedure, admission to the intensive care unit and length of stay were determined according to the patient’s clinical condition. The nasogastric tube (NGT) was removed on postoperative day 1 or 2, or when the volume of gastric output was less than 500 ml/day. In 10 patients, a nasogastric tube was not inserted. Total parenteral nutrition was used only in patients with grade C DGE or clinically relevant POPF. All patients were placed on proton pump inhibitor. In the majority of cases, erythromycin was administered intravenously at a dose of 250 mg every 6 h, which was converted to an oral formulation once an oral diet was tolerated. Serum amylase and drain-fluid amylase levels were measured on postoperative day 3. Drains were removed sequentially, if no evidence of pancreatic or bile leakage was present. Serum glucose level was maintained within normal limits by using an insulin sliding scale. When patients were clinically well and tolerated a solid diet, they were discharged from the hospital.

Delayed gastric emptying was defined according to the consensus definition proposed by the International Study Group of Pancreatic Surgery (ISGPS), using the web-based calculator (http://pancreasclub.com/calculator/). The severity of DGE was classified into 3 grades (A, B, or C) according to the ISGPS’s clinical criteria, based on the patient’s clinical course and postoperative management, such as the need for NGT in the postoperative period or the inability to tolerate solid oral intake. Grade A was defined as needing the NGT for more than 7 days or reinsertion of the NGT after postoperative day 3, or as being unable to tolerate a solid diet by postoperative day 7. Grade B was defined as needing for NGT for 8 to 14 days after surgery or reinsertion of the NGT after day 7, or as being unable to tolerate a solid diet by postoperative day 14. Grade C was defined as needing the NGT for more than 14 days or reinsertion of the NGT after day 14, or as being unable to tolerate a solid diet by day 21.

If evidence of DGE was present, then upper gastrointestinal contrast radiography was performed, and the time necessary for passage of the oral contrast agent from the esophagogastric junction into the jejunum was measured. Esophagogastroduodenoscopy (EGD) was performed to rule out mechanical obstruction of the GJA. A contrast computed tomography scan was performed if there was clinical evidence of infection contributing to DGE (i.e., fever, leukocytosis, and purulent drainage). Any suspicious intra-abdominal fluid collection that may have indicated an abscess was drained by interventional radiologists. The drainage fluid was sent for culture and amylase content analysis. The drains were removed when the fluid volume was <10 ml/day, the fluid amylase content was <3 times the upper limit of serum amylase, and the fluid collection was completely resolved, as determined by abdominal computed tomography scan.

### Data Analysis

Continuous variables were assessed for normality by using the Shapiro-Wilk test. For normally distributed variables, values were expressed as the mean ± standard deviation, and the difference between the two groups was tested by using the Student *t* test. For abnormally distributed variables, values were expressed as the median (range), and non-parametric tests (i.e., Wilcoxon rank-sum test, Mann-Whitney test) were used to assess the differences between the two groups. For categorical variables, the Pearson chi-square test or Fisher exact test was used for statistical comparison. All statistical tests were two tailed, and a *p* value of <0.05 was considered significant.

Univariate analysis was performed by using a non-conditional logistic regression model, and data were expressed as an odds ratio for each independent variable (potential risk factors). The odds ratio, 95 % confidence interval, and *p* value were calculated. Variables with a *p* value of <0.25 in the univariate analysis were included in the logistic regression model for the multivariate analysis. In the next step, variables with a *p* value of <0.1 in the multivariate analysis were included in the final logistic regression model. In the final model, a *p* value <0.05 was considered statistically significant.

## Results

Patient characteristics and preoperative data for group I (new technique) and group II (standard technique) patients are shown in Table 1. No significant differences were observed between the two groups with respect to age, sex, ASA grade, presence of comorbidities, or the number of patients who underwent preoperative endoscopic or percutaneous biliary drainage. Similarly, preoperative serum albumin and total bilirubin levels were comparable between the two groups. However, more patients in group I presented with preoperative diabetes than in group II (36.7 vs. 28.0 %, respectively; *p* = 0.045).

Intraoperative data for group I and group II patients are shown in Table 1. Data from both groups are comparable with respect to estimated amount of blood loss, blood transfusion, type of PD, concomitant procedures, and the rate of biliary infection. However, the median operative time was significantly longer in group II than in group I (*p* < 0.001).

Postoperative outcomes of group I and group II patients are shown in Table 2. Of the 208 patients, 6 (2.9 %) died within 90 days after PD. No significant difference was observed in the mortality rate between the two groups. Similarly, no significant difference was seen between the two groups in regard to the underlying disease or the 30-day readmission rate. However, the overall median postoperative length of hospital stay was significantly shorter in group I than in group II (*p* < 0.001). Similarly, the median blood glucose level was significantly lower in group I than in group II patients (*p* < 0.001). Ninety-one patients (43.8 %) developed one or more complications other than DGE. The incidence of postoperative complications other than DGE was significantly lower in group I than in group II (*p* = 0.03; Tables 2 and 3).

### Delayed Gastric Emptying

Six patients who died in the immediate postoperative period were excluded from the analysis because the cause of death was not related to DGE. Overall, 74/202 patients (36.7 %) developed DGE according to the ISGPS criteria (Table 4). The majority of patients had grade A (44/202; 21.8 %); 21 patients (10.4 %) had grade B, and 9 patients (4.5 %) had grade C DGE. The incidence of DGE was significantly lower in group I than in group II patients (*p* < 0.001). A total of 9 patients in group I (10.2 %) developed DGE, whereas 65 patients (57.0 %) in group II developed DGE. Significantly fewer patients in group I developed grade A, B, and C DGE than did patients in group II. In fact, none of the patients in group I had grade C DGE, whereas 9 patients (7.9 %) in group II did.

**Table 4:** Variables related to delayed gastric emptying (DGE) in group I and group II

Variable	Group I (*n* = 88)	Group II (*n* = 114)	*p* value
Overall postoperative DGE	9 (10.2 %)	65 (57.0 %)	<0.001*
Grade A	7 (8.0 %)	37 (32.5 %)	
Grade B	2 (2.3 %)	19 (16.7 %)	
Grade C	0	9 (7.9 %)	
Overall primary DGE	7 (8.0 %)	40 (35.1 %)	<0.001*
Grade A	6 (6.8 %)	30 (26.3 %)	
Grade B	1 (1.1 %)	6 (5.3 %)	
Grade C	0	4 (3.5 %)	
NGT removed on POD #	2 (1–18)	4.5 (2–16)	<0.001*
NGT reinserted anytime in the postoperative period	7 (8.0 %)	24 (21.1 %)	0.099
Able to tolerate solid food by POD #	4 (2–29)	7 (5–73)	<0.001*
Use of erythromycin	69 (78.4 %)	51 (44.7 %)	<0.001*
30-day readmission rate as a result of DGE	2 (2.3 %)	4 (3.5 %)	0.94

Data are expressed as the median (range) or as the number (percent)

*DGE* delayed gastric emptying, *NGT* nasogastric tube, *POD* postoperative day

*p < 0.05 was considered statistically significant

Compared to group II patients, group I patients had significantly shorter times to removal of the NGT and to starting a solid diet after surgery. However, no significant difference was observed between groups in the reinsertion rate of the NGT or in the 30-day readmission rate as a result of DGE. On the other hand, significantly more patients in group I than in group II received prophylactic intravenous erythromycin as a prokinetic agent. To determine the influence of erythromycin on the occurrence of DGE, we performed a subset analysis between patients with or without DGE. Although more patients with DGE received intravenous erythromycin than did patients without DGE, the difference was not statistically different (*p* = 0.69; Table 5).

**Table 5:** Univariate analysis of factors that may influence the development of delayed gastric emptying (DGE)

Variable	DGE		Odds ratio	95 % CI for OR	*p* value
	No (*n* = 128)	Yes (*n* = 74)			
Age (years)	59 (24–85)	65 (26–82)	1.0	1.0–1.1	0.046*
Sex					0.27
Male	62 (48.4 %)	37 (50.0 %)			
Female	71 (55.5 %)	37 (50.0 %)	0.7	0.4–1.3	
Diabetes mellitus	43 (33.5 %)	23 (31.1 %)	1.1	0.5–3.2	0.21*
Comorbidities	45 (35.2 %)	29 (39.2 %)	1.8	1.0–3.4	0.060*
ASA grade					0.033*
2	42 (32.8 %)	19 (25.7 %)			
3	72 (56.3 %)	43 (58.1 %)	1.3	0.7–2.6	
4	14 (10.9 %)	12 (16.2 %)	4.3	1.4–1.9	
Malignant tumors	96 (75.0 %)	53 (71.6 %)	1.0	0.5–1.9	0.91
ERCP/PTC	78 (60.9 %)	50 (67.6 %)	0.9	0.5–1.7	0.78
Serum albumin (g/dl)	4.2 (1.6–5.3)	4.1 (2.1–5)	0.7	0.4–1.3	0.28
Total bilirubin (mg/dl)	1.0 (0.2–18.3)	0.8 (0.2–29.7)	1.0	0.9–1.0	0.67
Orthotopic reconstruction	49 (38.3 %)	65 (87.8 %)	8.1	3.7–17.8	<0.001*
Surgery duration (min)	359 (227–609)	382 (246–885)	1.0	1.0–1.0	0.017*
Blood loss (ml)	300 (50–1300)	300 (50–3200)	1.0	1.0–1.0	0.38
Blood transfusion	24 (18.8 %)	19 (25.7 %)	1.3	0.6–2.7	0.38
Blood glucose (mg/dl)	140 (87–445)	154 (98–349)	1.0	1.0–1.0	0.031*
Erythromycin			1.0	0.3–3.2	0.69
Yes	72 (56.3 %)	45 (60.8 %)			
No	58 (45.3 %)	29 (39.2 %)			
POPF	7 (5.5 %)	10 (13.5 %)	2.8	1.0–7.5	0.042*
Other complications					<0.001*
No	106 (82.8 %)	43 (58.1 %)			
Yes	27 (21.1 %)	31 (41.9 %)	3.6	1.8–7.2	

*ASA* American Society of Anesthesiologists, *CI* confidence interval, *ERCP/PTC* endoscopic retrograde cholangiopancreatography/percutaneous transhepatic cholangiography, *OR* odds ratio, *POPF* postoperative pancreatic fistula

*p < 0.25 for these variables, which were therefore included in the multivariable analysis

In a separate analysis, other postoperative complications were excluded that may have affected the length of NGT placement and the patients’ tolerance of solid food, such as prolonged intubation, abdominal sepsis, and prolonged NPO for POPF. According to the ISGPS criteria, 47 patients (23.3 %) developed primary DGE. The rate of primary DGE was significantly lower in group I than in group II patients (8.0 vs. 35.1 %, respectively; *p* < 0.001). Furthermore, more patients in group II than in group I developed clinically relevant DGE (i.e., grades B and C). One patient in group I developed grade B DGE, whereas in group II, 6 patients developed grade B DGE and 4 patients developed grade C DGE (Table 4).

Univariate analysis of factors that may result in DGE revealed that older age, preoperative comorbidities, ASA grade 4, standard orthotopic technique, operative time, perioperative serum glucose level, and rate of complications including POPF were significant factors that influence the incidence of DGE. Variables with a *p* value <0.25 in the univariate analysis were included in the logistic regression model for the multivariate analysis. The multivariate analysis revealed that age, standard orthotopic technique, and postoperative rate of complications including POPF are independent risk factors for DGE (Table 6). The highest odds ratio was observed for the orthotopic standard technique (odds ratio = 8.5).

**Table 6:** Multivariate analysis of independent variables with the outcome of delayed gastric emptying (DGE)

Variables	Odds ratio	95 % CI for OR	*p* value
Age	1.0	1.0–1.1	0.022*
Orthotopic technique	8.5	3.7–19.7	<0.001*
POPF	3.7	1.1–12.6	0.044*
Complication	3.6	1.7–7.9	0.001*

*CI* confidence interval, *OR* odds ratio, *POPF* postoperative pancreatic fistula

*All of the odds ratios were significant at the alpha level of 0.05

## Discussion

We conducted a prospective cohort pilot study to evaluate the efficacy of preserving the gastric antrum with proximal Roux-en-y GJA in reducing the incidence of DGE after PD. In a group of 202 patients who underwent elective PD, we found that the rate of DGE and the length of hospital stay related to DGE were significantly lower in group I patients who underwent PD with the new technique than in group II patients who underwent PD with the standard orthotopic technique. In addition, more group II patients developed clinically relevant DGE (i.e., grades B and C), whereas only 2 patients (2.3 %) in group I developed grade B DGE, and no patients developed grade C DGE. Both patients in group I with grade B DGE developed intra-abdominal abscesses that contributed to generalized ileus and DGE.

The rates of overall and clinically relevant DGE in group II patients (57.0 and 24.6 %, respectively) were comparable to those observed in other studies.^1^–^19^,^33^,^42^,^43^ However, in patients treated with our new approach, the rates of overall and clinically relevant DGE were significantly reduced in comparison (10.2 and 2.3 %, respectively; *p* < 0.001). This could be interpreted as a result of the decreased complication rate seen in group I compared to group II patients, which corresponds to the decreased rate of POPF seen after June 2007, as shown previously.^41^ However, this improvement in the incidence of DGE also pertained to the rate of primary DGE when excluding postoperative complications other than DGE that influenced the length of NGT placement and the patients’ tolerance of solid food. Our findings corroborate those of a prospective, randomized, controlled study that reported a significant reduction in the incidence of overall and clinically relevant DGE in patients who underwent pylorus-resection PD with preservation of more than 95 % of the stomach when compared with patients who underwent pylorus-preserving PD (4.5 vs. 17.2 %, and 3 vs. 7.8 %, respectively).^33^ However, more patients in that study developed grade B and C DGE than in our study, as all of those patients underwent standard reconstruction of the GJA by using the antecolic approach.

We also found that the length of hospital stay as a result of DGE was longer in group II than in group I. Although an inverse relationship between length of hospital stay and readmission rate has been proposed,^44^,^45^ we observed no significant difference between groups in the 30-day readmission rate.

In various studies, several risk factors for DGE have been identified in univariate and multivariate analyses.^43^,^46^–^49^ In the present study, the results of our univariate analysis corroborated the findings of these studies. We found that the median operative time was longer in group II than in group I, despite an additional anastomosis that was performed in group I patients. The reason for this is not clear, given that the procedures differ only in the reconstruction technique, and all procedures were performed by three senior surgeons with more than 35 years of combined experience in hepatobiliary and pancreatic surgery. Although the length of the procedure was a predictor of DGE in univariate analysis, this was not confirmed in multivariate analysis. Additionally, we found that perioperative blood sugar was better controlled in group I than in group II. This may be attributed to a higher incidence of complications in group II than in group I. Although perioperative blood sugar level was a predictor for DGE in our univariate analysis, this was not confirmed in multivariate analysis. Our multivariate regression analysis showed that older age, severe postoperative complications including clinically relevant POPF, and standard orthotopic technique are independent risk factors for DGE. However, among the risk factors identified, the highest odds ratio was observed in the patients who underwent the standard technique.

Although self-limiting and non-fatal, the development of clinically relevant DGE leads to significant deviation from the normal clinical pathway and contributes significantly to the deterioration of the patient’s quality of life in the immediate postoperative period. Whereas primary DGE is caused by disruption of the neurohumoral pathway, several studies have shown that intra-abdominal complications and angulation and torsion of the gastrojejunostomy/duodenojejunostomy anastomosis are a possible cause of secondary DGE (54–59). In 1978, PpPD was developed by Traverso and Longmire^37^ to increase gastric volume and reduce complications related to gastric stasis and dumping syndrome. However, DGE still occurs in 30 to 50 % of patients who undergo PpPD.^17^,^35^ The mechanism for DGE after PpPD has been suggested to be multifactorial, including vascular and neural injury to the anteropyloric muscle,^31^,^32^ angulation of the duodenojejunostomy,^50^ and gastric atony after removal of the duodenum with diminished circulating motilin hormone.^29^

Despite numerous reports of case control and randomized controlled studies examining the antecolic vs. retrocolic route to the reconstruction of the gastrojejunal/duodenojejunal anastomosis, there is no consensus regarding which is the procedure of choice after PD.^10^,^16^,^34^,^51^–^53^ In all of these studies, regardless of the type of reconstruction (Billroth I or Billroth II), a lower incidence of DGE was observed in procedures that allowed for the straight passage of the gastric content under gravity into the jejunum. Although the use of Roux limbs after PD has been previously studied, our technique is unique in that we used the proximal 35–40 cm of the jejunum brought through the transverse mesocolon as an isolated Roux limb. This improved gastric emptying by providing a straight route for the gastric content to pass into the jejunum under gravity, avoiding the angulation or torsion of the GJA that has been previously reported in Billroth I retrocolic reconstruction.^53^

As stated above, motilin and its receptors play a significant role in regulating phase III of the MMC at the end of the postprandial period. Several studies have shown that resection of the duodenum after PpPD reduces the cyclic surge in plasma motilin levels, creating slow recovery of phase III with low amplitude in the efferent limb and slow propagation of the MMC downstream of the small intestine.^38^,^54^ Therefore, preserving the gastric antrum and using the proximal limb of the jejunum, which has a higher concentration of motilin receptors than does the distal limb, to reconstruct the GJA maximizes the utilization of motilin receptors in response to the transit of stomach content. This may, in part, compensate for the loss of duodenal secretions.

Erythromycin, a macrolide antibiotic, and derivatives of erythromycin have been shown to induce gastric phase III of the MMC in humans and dogs.^55^,^56^ Acting as a motilin agonist, it binds to motilin receptors. During the last two decades, the effects of erythromycin on gastrointestinal motility in gastrectomized patients have been studied extensively. A prospective, randomized, placebo-controlled, double-blinded study showed that erythromycin significantly improved gastric emptying in patients after PD.^19^ In the present study, we did not observe a significant effect of erythromycin on the incidence of DGE because more patients with DGE received erythromycin than did patients without DGE. However, the difference was not statistically significant (*p* = 0.69).

The drawbacks of this technique are the additional anastomosis required to establish continuity of the two loops of jejunum, which may negatively affect intestinal motility, and the difficulty of approaching the HJA endoscopically, if warranted. However, in our group I patients, we did not encounter any complications related to an additional jejunal anastomosis or the need for endoscopic examination of the HJA. In addition, a major limitation of this study is that it is a prospective cohort pilot study with potential type I error. To validate the results of this pilot study, we plan to conduct a randomized, controlled study.

In conclusion, we have demonstrated that the preservation of the gastric antrum with proximal Roux-en-y GJA after PD reduces the incidence of overall and clinically significant DGE, as well as the ensuing length of hospital stay. Notably, this effect was also observed in the subset of patients with primary DGE. Although this new approach dramatically reduced the incidence of DGE, it did not eliminate DGE completely, most likely because of the complexity of the procedure and the multiplicity of factors that are involved.
